# Evolutionary game analysis of community elderly care service regulation in the context of “Internet +”

**DOI:** 10.3389/fpubh.2022.1093451

**Published:** 2022-12-22

**Authors:** Qiangxiang Wang, June Liu, Yue Zheng

**Affiliations:** Department of Logistics and E-Commerce, School of Economics and Management, Huaibei Normal University, Huaibei, China

**Keywords:** community elderly care, feedback mechanism, service regulation, evolutionary game, system dynamics

## Abstract

**Background:**

As an upgraded form of the elderly care service industry, “Internet + Community Elderly Care” integrates information technology, artificial intelligence, Internet thinking, and the construction of community elderly care service mechanisms. Research on “Internet + Community Elderly Care” has become a focus.

**Methods:**

The four-party evolutionary game model of elderly service regulations was presented, which consists of the government, providers, platforms, and elderly people. By using Lyapunov stability theory, the stability of each player's strategy selection was analyzed. Furthermore, the sensitivity analysis of the key parameters was discussed in detail using system dynamics.

**Results and discussion:**

Online evaluations of elderly people have more positive effects on the regulatory system than offline evaluations. Both the penalties on providers and subsidies on platforms given by the government have thresholds. Moreover, government penalties for providers and subsidies for platforms could curb their speculative behavior and enable effective steering of providers and platforms.

**Conclusion:**

The Omni-feedback mechanism for elderly people can effectively curb the speculative behavior of elderly care service providers and elderly care service information platforms. The government should dynamically adjust penalties and subsidy policies.

## 1. Introduction

Community elderly care can reduce the financial burden associated with aging (1) and meet the demands of the elderly in their homes (2–4). Therefore, community elderly care is an important model for assisting elderly people in adapting to the trend of social development and improving their quality of life in their later years. However, with the issue of an aging population becoming serious in some countries, including China, the drawbacks of the traditional community-based elderly care model have gradually emerged. For example, a lack of talents and professionalism (5–7), poor accessibility of services and single service item (5, 7, 8), difficult quality measurement, a lackluster supervision and evaluation system (9), inefficient management, and a mismatch between supply and demand (5, 10, 11).

In recent years, with the development and proliferation of “Internet +” information technology, the mode of combining “Internet +” with the elderly care industry has gradually become a focus (12). The combination of “Internet +” and elderly care can better match the supply and demand of elderly care services, optimize the integration and allocation, promote the specialization, intelligence, and standardization of the elderly care services, and improve the service quality of elderly care (5, 13). Thus, the combination of “Internet +” and the elderly care industry has received the attention and support of governments in various countries. For example, Cherie and Sajda (14) pointed out that the US has been seeking to apply information technology to elderly care services and form a network of smart elderly care services. Schnell (15) introduced the relevant situation of smart health care in Japan, and the “Tokyo Model” smart elderly care community was created in Tokyo, Japan.

Similarly, in China, the government has issued a series of policies to encourage the development of new elderly care, especially “Internet + Community Elderly Care.” In December 2016, China's State Council issued “Several Opinions on Fully Opening the Elderly Care Service Market and Improving the Quality of Elderly Care Service” (16), which promotes the integration of information technology, such as mobile Internet, with the elderly services industry. In April 2019, China's State Council issued “Opinions on Promoting the Development of Elderly Care Services,” proposing to implement the “Internet + Elderly Care” action (17). In February 2022, China's State Council issued the “14th Five-Year Plan for the Development of the National Aging Cause and Elderly Care Service System” (18), which promotes Internet platform enterprises to match the demand for elderly care services and support the platform-based display of community elderly care service institutions. These proposed policies will promote “Internet+” elderly care service innovation. However, there are some problems with the practice of “Internet + Community Elderly Care.” For example, some conflicts of interest among stakeholders may exist, the division of powers and responsibilities may not be clear, and there may be a lack of both online supervision and offline service tracking on the platforms providing information on elderly care services. These would lead to a lack of effective supervision of the elderly care service providers' service provision practices, which encourages the speculative behavior of elderly care service providers to provide substandard services. Therefore, the relationship among government departments, elderly care service providers, elderly care service information platforms, and elderly people should be properly discussed. Furthermore, the path of upgrading government policies should be explored to standardize the provider behavior of elderly care service providers and promote the healthy development of the “Internet + Community Elderly Care” industry.

Notably, the existing research on community elderly care is mainly focused on the concept of community elderly care (19), the demand for community elderly care (20, 21), the technology of community elderly care (22–25), and the model of community elderly care (1). However, there is little research on the quality of service in community elderly care. It is common knowledge that a strict regulatory system is conducive to the sustainable development of the elderly care industry (26). Based on this, some authors not only discussed how to improve the quality of community elderly care services, but also focused on improving the supervision of elderly care services. For example, Xu et al. (9) showed that the perception of the elderly in multi-attribute decision-making is ambiguous and established an effective evaluation method for the quality of intelligent community elderly services. Shao et al. (11) developed a model to screen performance optimization directions through sensitivity analysis conducive to the sustainable development of the community elderly care service system. Wang et al. (27) presented the problems in community elderly care through semi-structured interviews and provided some recommendations, including establishing a platform for government-citizen dialogue and establishing a sound monitoring and evaluation mechanism. Jiang et al. (28) analyzed the impact of government subsidies on the willingness of enterprises to provide high-quality services. Wang and Cui (29) discussed the impact of dynamic reward or penalty mechanisms on the self-discipline behavior of elderly care institutions. Under positive government regulations, Yue and Lin (30) pointed out that increased penalties can curb the speculation of service providers. Otherwise, penalties imposed by the government will be ineffective.

The above research is mainly based on the dominant role of government, with less consideration given to other subjects in the elderly service system. In supervising elderly services, government regulations can effectively improve the service quality of elderly care institutions (31). For “Internet + Community Elderly Care,” the government needs to allocate more resources to arranging, planning, and regulating the supply of community elderly care services. The overall planning capacity of the government may be inadequate. Consumer feedback also has an impact on product quality and corporate behavior. For example, He et al. (32) pointed out that, as end users, consumers have the right to provide feedback on product quality. Chevalier et al. (33) showed that consumer feedback evaluation has an impact on product quality. Yang et al. (34) concluded that consumers' feedback evaluations would affect potential customers' purchase decisions. Zheng et al. (35) believed that the online reputation mechanism based on feedback could inhibit the speculative behavior of enterprises. Zhang et al. (36) analyzed the development of e-commerce platforms and logistics enterprise strategies in response to consumer complaints. He et al. (37) studied the impact of consumer feedback on green product quality supervision by integrating the feedback channels of consumer online evaluation and complaints. The above research is based mainly on the feedback of consumers' online evaluations and complaints. In “Internet + Community Elderly Care,” offline evaluation and return visits are important feedback channels for elderly people. It is of great theoretical significance and practical value to integrate the feedback channels of online-offline evaluation, complaint, and return visits of the elderly and to develop an omnichannel feedback mechanism for the elderly.

In summary, most research is focused mainly on the concept, demand, technology, and model of community elderly care, with inadequate attention paid to the regulation of the service quality of “Internet + Community Elderly Care,” especially the elderly people's participation in the supervision system of elderly care services. In the process of the “Internet + Community Elderly Care” service supply, the elderly are not only the end consumers of services but also play the role of supervisors. Their supervisory role should not be ignored. Therefore, in this study, we used evolutionary game theory and system dynamics to focus on the relationship between the government, providers, platforms, and elderly people, aiming to solve the following two questions: (1) What is the impact of Omni-feedback mechanism on the elderly care service providers and elderly care service information platforms' behavior? (2) What is the impact of government penalties and subsidies on elderly care service providers and elderly care service information platforms' behavior? These findings will help the government better understand the elderly's evaluation behavior and provide beneficial enlightenment for formulating dynamic adjustment penalties and subsidy policies. Based on these reasons, the contributions of the study are proposed in the following three aspects:

1. The omnichannel feedback mechanism of the elderly in the “Internet + Community Elderly Care” regulatory system is presented. The relationship between the feedback of the elderly and the strategic choices of all players is deeply analyzed.
2. Some relevant parameters, such as the size of the offline social network, reputation, and service utility, are introduced in the proposed model. Furthermore, the impact of these parameters on the choice of behavior strategies of each player is quantified.
3. From the perspective of the whole system, a four-party evolutionary game model is constructed, and its corresponding system dynamics model is also proposed. It is conducive to analyzing each player's dynamic behavior, choice, and interaction to obtain more scientific and valuable conclusions.

## 2. Problem description and assumptions

### 2.1. Problem description

**Figure 1:**
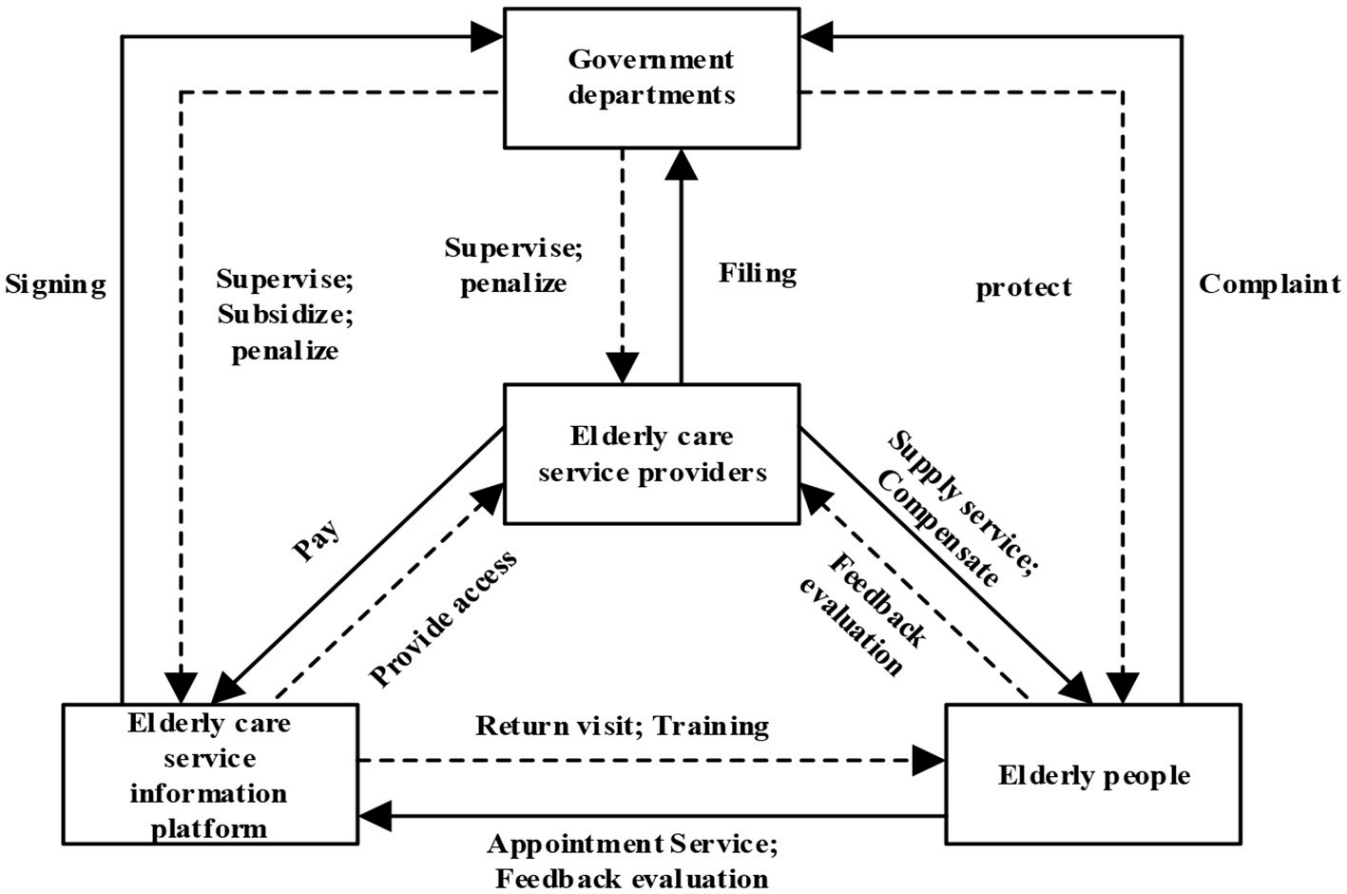
The relationships among the various subjects.

It is common knowledge that speculative behavior among elderly care service providers has become an important issue that cannot be ignored in “Internet + Community Elderly Care.” The omnichannel feedback mechanism of the elderly and the government's regulatory role can inhibit the speculative behavior of elderly care service providers. The omnichannel feedback mechanism includes the online evaluation conducted by the online evaluation system after the elderly purchase services through online channels and the offline evaluation conducted by the social network after the elderly purchase services through offline channels. Moreover, the return visits of the elderly care service information platforms and the complaint behaviors of the elderly are also part of the content of the Omni-feedback mechanism. The government can restrict the behavior of elderly care service providers and elderly care service information platforms through the policy adjustment of regulations, subsidies, and penalties. These policy adjustments can guide the healthy development of the “Internet + Community Elderly Care” industry. The relationships between the various subjects in “Internet + Community Elderly Care” are shown in Figure 1.

### 2.2. Model assumptions

This study used the evolutionary game as a research method because the regulation of “Internet + Community Elderly Care” services often involves multiple stakeholders with limited rationality, and the strategic choice of each stakeholder is influenced by many factors such as income, cost, and mutual influence. Stakeholders interact with each other through multiple rounds of strategy selection to achieve a stable state of strategy selection, which is the same as the characteristics of evolutionary game theory (EGT) (38). At present, EGT has been successfully applied to various economic and social issues, such as environmental governance (39–42), supply chain collaboration (43, 44), green building development (45, 46), online car-hailing platform regulations (47–49) and elderly service provision (50, 51). Therefore, EGT is more suitable for us to study the regulation of “Internet + Community Elderly Care” services.

The following hypotheses are proposed to construct the four-party evolutionary game model.

**Hypothesis 1**. Government departments, elderly care service providers, elderly care service information platforms, and elderly people are selected as the subjects of the four-party game. The probability of elderly care service providers offering high-quality (H.Q.) or low-quality service (L.Q.) is (*x*, 1 − *x*). The probability of the elderly care service information platforms performing positive return visits (P.V.) or negative return visits (N.V.) is (*y*, 1 − *y*). The probability of government departments enforcing positive regulations (P.R.) or negative regulations (N.R.) is (*z*, 1 − *z*). The probability that elderly people choose online evaluation (ON) or offline evaluation (OF) toward the services they have purchased is (*w*, 1 − *w*). Here, *x, y, z, w* ∈ [0, 1].

**Hypothesis 2**. The elderly care service providers can earn (1 − α)*Rs* for services offered through the elderly care service information platforms channel, where *Rs* is the total revenue of both the elderly care service providers and information platforms, α is the percentage of revenue to the elderly care service information platforms. The cost of providing high-quality service to elderly people is *Csh*, and the cost of providing low-quality service is *Csl*(*Csh* > *Csl*). When the elderly care service providers provide low-quality services, they will be subjected to administrative penalties from the government and are required to compensate the elderly who purchased the service for *Io*.

**Hypothesis 3**. The elderly care service information platforms make return visits to customers who purchase and use elderly care services at the cost of *Cei*. When the elderly care service providers publish their service information through the elderly care service information platforms, elderly care service information platforms will collect α*Rs* as commission income. The operating cost of the elderly care service information platforms is *Ceo*. When the elderly care service information platforms return positively, they will receive operating subsidies *S* from government departments. When the elderly care service information platforms return negatively and the elderly care service providers are confirmed to be providing low-quality services, they will be fined *Fe* by the government.

**Hypothesis 4**. The cost of positive regulations by the government is *Cg*. When the government imposes regulations negatively, the elderly may file a complaint, which may result in the government being fined by its superior government with penalties *Fg*. When offering high-quality services, elderly care service providers will produce social welfare *Rg*. When providing low-quality services, elderly care service providers may hurt the interests and even the health of the elderly. It would disrupt the development of the “Internet + Community Elderly Care” industry and cause social loss *Dg*.

**Hypothesis 5**. When providing online evaluations, the elderly will make some effort, such as learning the software for making appointments, evaluating, and spending some time, which costs *Cw*. On the other hand, offline evaluations incur *Cm* for the use of a smart terminal calling device such as a push-to-talk or phone. The elderly care service providers provide high-quality services that will bring physical and mental pleasure to the elderly, generating gainful utility *Ro*. On the contrary, the low-quality services provided by elderly care service providers will cause financial, physical, and mental damage *Do* to elderly people. When their rights are violated, the elderly may have a certain probability of complaining to the relevant government to safeguard their rights, with a rate of β, β ∈ [0, 1], and a cost of *Co*.

**Hypothesis 6**. The size of the offline social network for elderly people is γ ∈ [0, 1], which indicates the proportion of the potential customer affected by offline evaluations compared to the potential customer affected by online evaluations. In the omnichannel feedback mechanism, the elderly care service providers who provide high-quality services will gain the trust of elderly people and their families. They may gain more new clients and then have a reputational gain *Is*. The elderly care service providers who offer low-quality services will suffer reputational losses *Ds* due to damage to their reputation and loss of market share. Similarly, positive return visits by the elderly care service information platforms will promote reputational gains *Ie*, while negative ones will cause reputational losses *De*.

## 3. Stability analysis of players' strategy choices

Based on Hypotheses 1–5 in Section 2, a game payment matrix of the government, elderly care service providers, elderly care service information platforms, and the elderly are constructed under different strategy choices, which are shown in Table 1.

**Table 1:** Payment matrix for the players.

**Elderly care service information platforms**			**Government**			
			**PR (** ***z*** **)**		**NR (1 –** ***z*****)**	
**Elderly people**			**ON (** ***w*** **)**	**OF (1 –** ***w*****)**	**ON (** ***w*** **)**	**OF (1 –** ***w*****)**
**Elderly care service providers**	**HQ (** ***x*** **)**	**PV (y)**	(1 − α)*Rs* − *Csh* + *Is*, α*Rs* − *Cei* − *Ceo* + *S* + *Ie*, *Rg* − *Cg* − *S*, *Ro* − *Cw*	(1 − α)*Rs* − *Csh* + *Isγ*, α*Rs* − *Cei* − *Ceo* + *S* + *Ieγ*, *Rg* − *Cg* − *S*, *Ro* − *Cm*	(1 − α)*Rs* − *Csh* + *Is*, α*Rs* − *Cei* − *Ceo* + *S* + *Ie*, *Rg* − *S*, *Ro* − *Cw*	(1 − α)*Rs* − *Csh* + *Isγ*, α*Rs* − *Cei* − *Ceo* + *S* + *Ieγ*, *Rg* − *S*, *Ro* − *Cm*
		**NV** (1 − *y*)	(1-α)*Rs* − *Csh* + *Is*, α*Rs* − *Ceo* − *De*, *Rg* − *Cg*, *Ro* − *Cw*	(1-α)*Rs* − *Csh* + *Isγ*, α*Rs* − *Ceo* − *Deγ*, *Rg* − *Cg*, *Ro* − *Cm*	(1-α)*Rs* − *Csh* + *Is*, α*Rs* − *Ceo* − *De*, *Rg*, *Ro* − *Cw*	(1-α)*Rs* − *Csh* + *Isγ*, α*Rs* − *Ceo* − *Deγ*, *Rg*, *Ro* − *Cm*
	**LQ** (1 − *x*)	**PV** (*y*)	(1 − α)*Rs* − *Csl* − *Io* − *Fs* − *Ds*, α*Rs* − *Cei* − *Ceo* + *S* + *Ie*, *Fs* − *S* − *Cg* − *Dg*, *Io* − *Cw* − *Do*	(1 − α)*Rs* − *Csl* − *Io* − *Fs* − *Dsγ*, α*Rs* − *Cei* − *Ceo* + *S* + *Ieγ*, *Fs* − *S* − *Cg* − *Dg*, *Io* − *Cm* − *Do*	(1 − α)*Rs* − *Csl* − *Ds* − *Io*, α*Rs* − *Cei* − *Ceo* + *S* + *Ie*, −*S* − *Dg*, *Io* − *Cw* − *Do*	(1 − α)*Rs* − *Csl* − *Dsγ* − *Io*, α*Rs* − *Cei* − *Ceo* + *S* + *Ieγ*, −*S* − *Dg*, *Io* − *Cm* − *Do*
		**NV**(1 − *y*)	(1 − α)*Rs* − *Csl* − *Io* − *Fs* − *Ds*, α*Rs* − *Ceo* − *Fe* − *De*, *Fs* + *Fe* − *Cg* − *Dg*, *Io* − *Cw* − *Do*	(1 − α)*Rs* − *Csl* − *Io* − *Fs* − *Dsγ*, α*Rs* − *Ceo* − *Fe* − *Deγ*, *Fs* + *Fe* − *Cg* − *Dg*, *Io* − *Cm* − *Do*	(1 − α)*Rs* − *Csl* − β*Fs* − *Ds* − β*Io*, α*Rs* − *Ceo* − β*Fe* − *De*, β(*Fs* + *Fe* − *Fg*)−*Dg*, β(*Io* − *Co*)−*Cw* − *Do*	(1 − α)*Rs* − *Csl* − β*Fs* − *Dsγ* − β*Io*, α*Rs* − *Ceo* − β*Fe* − *Deγ*, β(*Fs* + *Fe* − *Fg*)−*Dg*, β(*Io* − *Co*)−*Cm* − *Do*

### 3.1. Stability analysis for elderly care service providers

It follows from the game payment matrix that the expected revenue of the elderly care service providers who choose to provide high-quality services (low-quality services) is *Ux*(*U*_1 − *x*_), and the average expected revenue of the elderly care service providers is Ū*x*:

(1) $$\begin{array}{l} Ux=\left(1-\alpha \right)Rs-Csh+wIs+\left(1-w\right)Is\gamma \end{array}$$

(2) $$\begin{array}{l} U_{1-x}=\left(1-\alpha \right)Rs-Csl-wDs-\left(1-w\right)Ds\gamma -zFs\\ \;-yIo-\left(1-y\right)zIo-\left(1-y\right)\left(1-z\right)\left(\beta Fs+\beta Io\right) \end{array}$$

(3) $$\begin{array}{l} Ūx=xUx+\left(1-x\right)U_{1-x} \end{array}$$

By using the Malthusian equation, the replicator dynamic equation and the first-order derivative of the elderly care service providers can be written as follows:

(4) $$\begin{array}{l} F\left(x\right)=dx/dt=x\left(Ux-Ūx\right)=x\left(1-x\right)G\left(y,z,w\right) \end{array}$$

(5) $$\begin{array}{l} F^{\prime }\left(x\right)=\left(1-2x\right)G\left(y,z,w\right) \end{array}$$

(6) $$\begin{array}{l} G\left(y,z,w\right)=w\left(1-\gamma \right)\left(I_{s}+D_{s}\right)+zF_{s}+C_{sl}-C_{sh}+I_{s}\gamma +D_{s}\gamma \\ \;+yI_{o}+\left(1-y\right)zI_{o}+\left(1-y\right)\left(1-z\right)\left(\beta F_{s}+\beta I_{o}\right) \end{array}$$

The dynamic replicator equation shows that some factors influence the service provision of elderly care service providers, such as the strategic choice of other players and all other factors that are closely related to the cost-benefit of the elderly care service providers. Using the stability theorem of the differential equation, the probability of decision-making of the elderly care service providers in a stable state must satisfy *F*(*x*) = 0 and *F*′(*x*) < 0.

**Proposition 1**. When *w* > *w*_0_, elderly care service providers provide a high-quality service. When *w* < *w*_0_, elderly care service providers provide low-quality service. When *w* = *w*_0_, its stabilization strategy cannot be determined. The threshold *w*_0_ is defined as follows:

$$\begin{array}{l} w_{o}=\{C_{sh}-C_{sl}-I_{s}\gamma -D_{s}\gamma -zF_{s}-yI_{o}-\left(1-y\right)[\left(1-z\right)(\beta F_{s}\\ \;+\beta I_{o})+z_{o}I]\}/\left(1-y\right)\left(I_{s}+D_{s}\right). \end{array}$$

**Proof of Proposition 1**. Because ∂*G*(*y, z, w*)/∂*w* > 0 *G*(*y, z, w*) is an increasing function of *w*. When *w* < *w*_0_, we have that *G*(*y, z, w*) < 0, *F*(*x*)|_*x* = 0_ = 0, $F^{\prime }\left(x\right){|}_{x=0}<0$, and then *x* = 0 has stability. When *w* > *w*_0_, *G*(*y, z, w*) > 0, *F*(*x*)|_*x* = 1_ = 0, $F^{\prime }\left(x\right){|}_{x=1}<0$, and then *x* = 1 has stability. When *w* = *w*_0_, *G*(*y, z, w*) = 0, *F*(*x*) = 0 and *F*′(*x*) = 0, which cannot determine a stable strategy. The proof of Proposition 1 is complete.

Proposition 1 suggests that an increase in the probability of online evaluations by elderly people would lead to a shift in the stabilization strategy of elderly care service providers from providing low-quality services to supplying high-quality services. However, an increase in the proportion of offline evaluations by elderly people will make the elderly care service providers take risks and eventually choose to provide low-quality services. This implies that the online evaluation behavior of elderly people can be an effective deterrent to the supply of low-quality services by elderly service providers. The elderly should be motivated to take the initiative and actively learn how to operate the software for elderly services.

**Figure 2:**
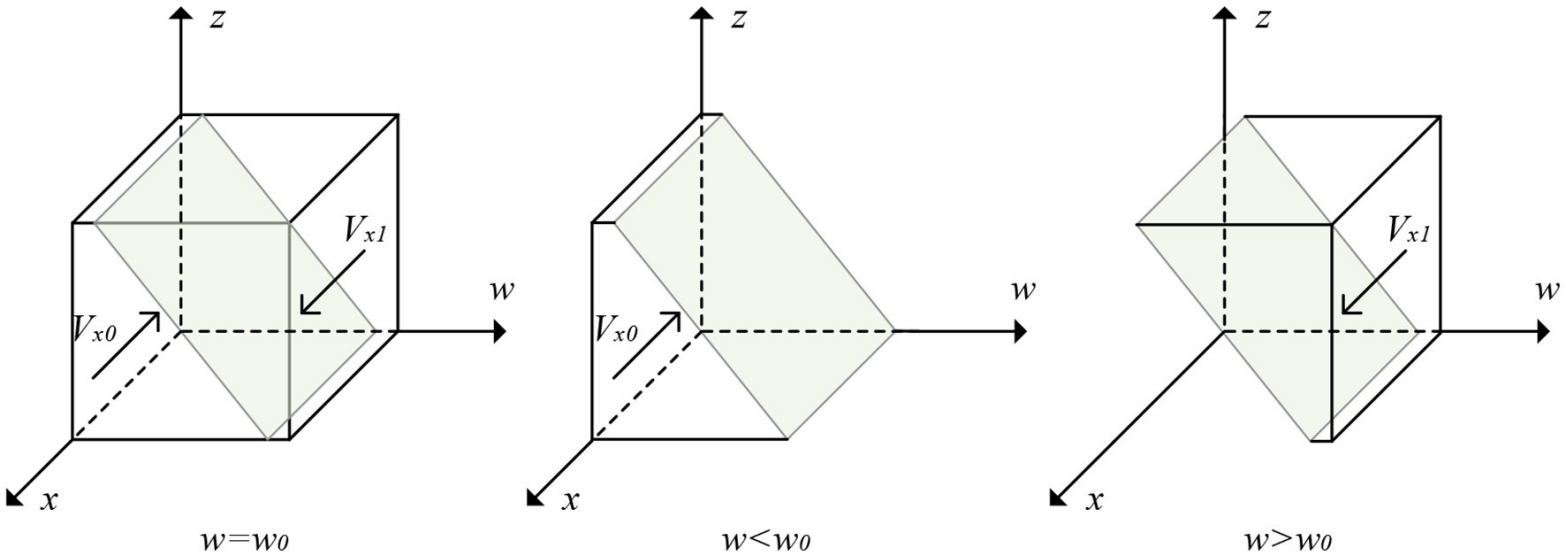
The evolutionary trajectory of elderly care service providers' strategy.

From Proposition 1, the evolutionary trajectory of elderly care service providers' strategies is obtained, as shown in Figure 2.

From Figure 2, the volume of part *Vx*0 is the probability that the elderly care service providers will choose to provide low-quality services. The volume of part *Vx*1 is the probability that it will supply high-quality services. Let *a* = *Csh* − *Csl* − *Isγ* − *Dsγ* − *yIo* − β(1 − *y*)(*Fs* + *Io*) and *b* = (1 − *y*)(β*Fs* + β*Io* − *Io*)−*Fs*. Then *w*_0_ = (*a* + *bz*)/(1 − γ)(*Is* + *Ds*), it is easy to obtain the following:

(7) $$\begin{array}{l} Vx0=\int_{0}^{1}\int_{0}^{1}\left(a+bz\right)/\left(1-\gamma \right)\left(I_{s}+D_{s}\right)dzdx\\ \;=\left(2a+b\right)/2\left(1-\gamma \right)\left(I_{s}+D_{s}\right) \end{array}$$

(8) $$\begin{array}{l} Vx1=1-Vx0=1-\left(2a+b\right)/2\left(1-\gamma \right)\left(Is+Ds\right) \end{array}$$

**Corollary 1.1**. The elderly care service providers will be more inclined to provide low-quality services when there is an increase in cost shrinkage arising from the provision of low-quality services. On the contrary, when reputational gains or losses increase, or when penalties for illegal acts of the elderly care service platforms increase, the elderly care service providers will be more inclined to provide high-quality services.

**Proof of Corollary 1.1**. The first order derivative from *Vx*1 for *Csh* − *Csl*, *Is*, *Ds*, *Fs*, respectively, gives the following:

$$\begin{array}{l} \partial Vx1/\partial \left(Csh-Csl\right)=-1/\left(1-\gamma \right)\left(Is+Ds\right)<0, \end{array}$$

$$\begin{array}{l} \partial Vx1/\partial Is=\left[\gamma \left(1-\gamma \right)\left(Is+Ds\right)+\left(1-\gamma \right)\left(2a+b\right)\right]\\ \;/2{\left(1-\gamma \right)}^{2}{\left(Is+Ds\right)}^{2}>0, \end{array}$$

$$\begin{array}{l} \partial Vx1/\partial Ds=\left[\gamma \left(1-\gamma \right)\left(Is+Ds\right)+\left(1-\gamma \right)\left(2a+b\right)\right]\\ \;/2{\left(1-\gamma \right)}^{2}{\left(Is+Ds\right)}^{2}>0, \end{array}$$

$$\begin{array}{l} \partial Vx1/\partial Fs=\left(2\beta -2\beta y-\beta z+\beta yz+z\right)\\ \;/2\left(1-\gamma \right)\left(Is+Ds\right)>0. \end{array}$$

The proof of Corollary 1.1 is complete.

**Corollary 1.2**. Government should impose penalties *Fs* greater than a certain threshold *F*′*s* to ensure that elderly care service providers provide high-quality services. The following scenarios may occur. For example, the gains of low-quality services in illegal increase; the reputational gains and losses decrease; the likelihood of online evaluation decline; the size of offline social networks decreases; compensations decrease; and the number of complaints from elderly people decreases. Under these scenarios, the government needs to increase fines. Here, the threshold *F*′*s* is defined as follows:

$$\begin{array}{l} F^{\prime }s=\{Csh-Csl-w\left(1-\gamma \right)\left(Is+Ds\right)-Is\gamma -Ds\gamma -yIo\\ \;-\left(1-y\right)\left[\left(1-z\right)\beta Io+zIo\right]\}/[z+\left(1-y\right)\left(1-z\right)\beta ]. \end{array}$$

**Proof of Corollary 1.2**. It follows from $F^{\prime }\left(x\right){|}_{x=1}<0$ and Proposition 1 that *Fs* > *F*′*s*. By calculating the first-order partial derivative of *Csh* − *Csl*, *Is*, *Ds*, *w*, γ, *Io*, and β for *F*′*s*, respectively, it is easy to conclude that ∂*F*′*s*/∂(*Csh* − *Csl*) > 0, ∂*F*′*s*/∂*Is* < 0, ∂*F*′*s*/∂*Ds* < 0, ∂*F*′*s*/∂*w* < 0, ∂*F*′*s*/∂γ < 0, ∂*F*′*s*/∂*Io* < 0 and ∂*F*′*s*/∂β < 0. Therefore, *F*′*s* is positively correlated with *Csh* − *Csl* and negatively correlated with *Is*, *Ds*, *w*, γ, *Io* and β, respectively. The proof of Corollary 1.2 is complete.

### 3.2. Stability analysis for elderly care service information platforms

The expected revenue for the positive return visit (negative return visit) strategy chosen by the elderly care service information platforms is *Uy*(*U*_1 − *y*_), and its average expected revenue is Ū*y*:

(9) $$\begin{array}{l} Uy=\alpha Rs-Ceo-Cei+wIe+\left(1-w\right)Ie\gamma +S \end{array}$$

(10) $$\begin{array}{l} U_{1-y}=\alpha Rs-Ceo-wDe-\left(1-w\right)De\gamma \\ \;-\left(1-x\right)\left[zFe+\left(1-z\right)\beta Fe\right] \end{array}$$

(11) $$\begin{array}{l} Ūy=yUy+\left(1-y\right)U_{1-y} \end{array}$$

The replicator dynamic equation and first-order derivative of the elderly care service information platforms can be calculated as follows:

(12) $$\begin{array}{l} F\left(y\right)=dy/dt=y\left(Uy-Ūy\right)=y\left(1-y\right)H\left(x,z,w\right) \end{array}$$

(13) $$\begin{array}{l} F^{\prime }\left(y\right)=\left(1-2y\right)H\left(x,z,w\right) \end{array}$$

(14) $$\begin{array}{l} H\left(x,z,w\right)=w\left(1-\gamma \right)\left(Ie+De\right)+Ie\gamma +De\gamma \\ \;+S-Cei+\left(1-x\right)\left[zFe+\left(1-z\right)\beta Fe\right] \end{array}$$

From Equations 12–14, it can be seen that the strategy of the elderly care service information platforms mainly depends on the choices of the three remaining parties, the strength of the government penalties or subsidies, and the size of reputational gains and losses. By using the stability theorem of a differential equation, the decision probability of the elderly care service information platforms in a stable state must satisfy *F*(*y*) = 0 and *F*′(*y*) < 0.

**Proposition 2**. When *w* > *w*_1_, the elderly care service information platforms choose positive return visits. When *w* < *w*_1_, the elderly care service information platforms will choose negative return visits. When *w* = *w*_1_, its stabilization strategy cannot be determined. The threshold *w*1 is defined as follows:

$$\begin{array}{l} w_{1}=\;\{\;Cei-S-Ie\gamma -De\gamma \\ \;-\left(1-x\right)\left[zFe+\left(1-z\right)\beta Fe\right]\;\}\;/\left(1-\gamma \right)\left(Ie+De\right). \end{array}$$

**Proof of Proposition 2**. Since ∂*H*(*x, z, w*)/∂*w* > 0, *H*(*x, z, w*) is an increasing function with regard to *w*. When *w* < *w*_1_, we have that *H*(*x, z, w*) < 0, *F*(*y*)|_*y* = 0_ = 0, $F^{\prime }\left(y\right){|}_{y\;=\;0}<0$, and then *y* = 0 has stability. When *w* > *w*_1_, *H*(*x, z, w*) > 0, *F*(*y*)|_*y* = 1_ = 0, $F^{\prime }\left(y\right){|}_{y\;=\;1}<0$, and then *y* = 1 has stability. When *w* = *w*_1_, we can obtain that *H*(*x, z, w*) = 0, and then *F*(*y*) = 0 and *F*′(*y*) = 0. Now, no stable strategy can be identified. The proof of Proposition 2 is complete.

It follows from Proposition 2 that an increase in the probability of online evaluation by elderly people will make the elderly care service information platforms more inclined to choose positive return visits. In contrast, an increase in the proportion of elderly people who evaluate offline will reduce both the possible reputational gains and losses faced by elderly care service information platforms. With the lower self-regulatory business gains and higher speculative business gains, the strategy of elderly care service information platforms will shift from positive return visits to negative return visits.

**Figure 3:**
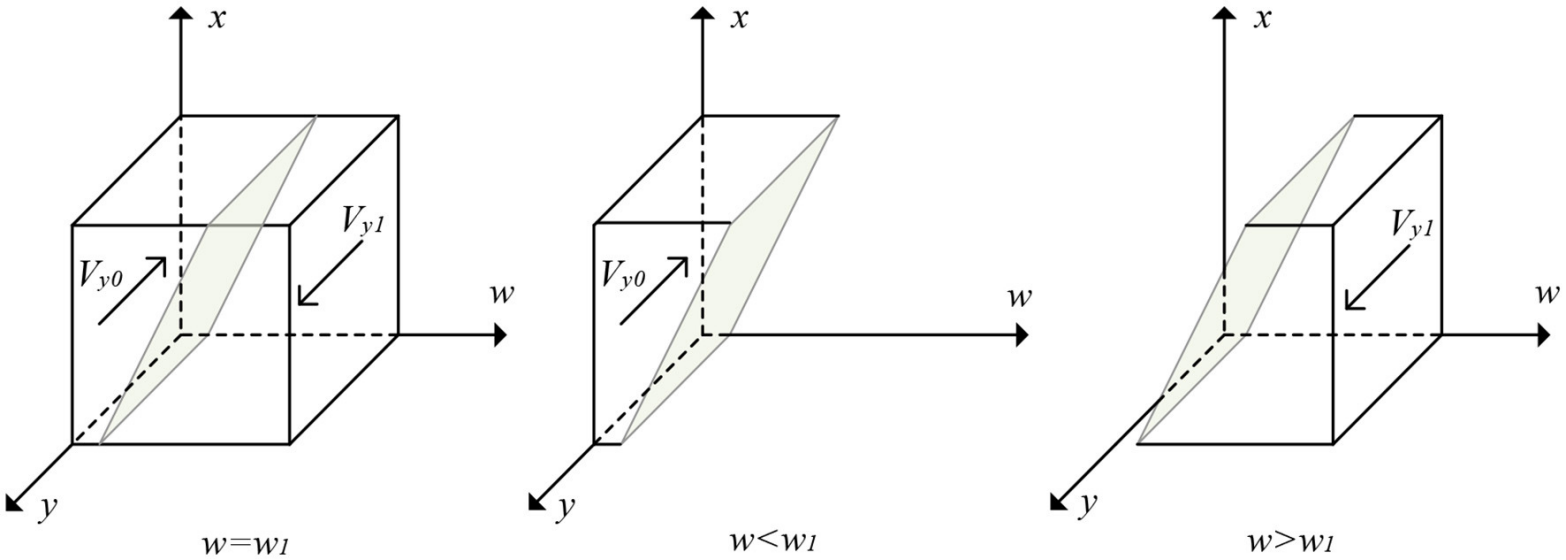
The evolutionary trajectory of elderly care service information platforms' strategy.

From Proposition 2, the evolutionary trajectory of elderly care service information platforms' strategy is obtained, as shown in Figure 3.

Figure 3 shows the volumes of parts *Vy*0 and *Vy*1 are the probabilities of the elderly care service information platforms choosing negative return visits and positive return visits, respectively. Let *c* = *Cei* − *S* − *Ieγ* − *Deγ* and *d* = *zFe* + (1 − *z*)β*Fe*. Then, *w*_1_ = (*c* − *d* + *dx*)/(1 − γ)(*Ie* + *De*), and we have the following equation:

(15) $$\begin{array}{l} Vy0=\int_{0}^{1}\int_{0}^{1}\left(c-d+dx\right)/\left(1-\gamma \right)\left(Ie+De\right)dxdy\\ \;=\left(2c-d\right)/2\left(1-\gamma \right)\left(Ie+De\right) \end{array}$$

(16) $$\begin{array}{l} Vy1=1-Vy0=1-\left(2c-d\right)/2\left(1-\gamma \right)\left(Ie+De\right) \end{array}$$

**Corollary 2.1**. When the cost of positive return visits by elderly care service information platforms increases, those platforms will be more inclined to a negative return visit. When reputational gains or losses increase, the complaint rate of elderly people increases, the government imposes penalties, and the elderly care service information platforms will be more inclined to make positive return visits.

**Proof of Corollary 2.1**. The first order derivative from *Vy*1 for *Cei*, β, *Ie*, *De* and *Fe*, respectively, gives ∂*Vy*1/∂*Cei* < 0, ∂*Vy*1/∂β > 0, ∂*Vy*1/∂*Ie* > 0, ∂*Vy*1/∂*De* > 0, ∂*Vy*1/∂*Fe* > 0. The proof of Corollary 2.1 is complete.

**Corollary 2.2**. The government subsidies *S* to the elderly care service information platforms should be greater than a certain threshold *S*′ to effectively use these platforms. When some issues emerge, the government should increase policy subsidies for elderly care service information platforms. These scenarios include an increase in the cost of return visits, a decrease in the probability of online evaluations, a decrease in reputational gains and losses, a decrease in the size of offline social networks, an increase in the probability of providing high-quality services, a decrease in the intensity of government supervision and punishment, and a decrease in the complaint rate among the elderly. Here, the threshold *S*′ is defined as follows:

$$\begin{array}{l} S^{\prime }=Cei-Ie\gamma -De\gamma -w\left(1-\gamma \right)\left(Ie+De\right)\\ \;-\left(1-x\right)\left[zFe+\left(1-z\right)\beta Fe\right]. \end{array}$$

**Proof of Corollary 2.2**. It follows from $F^{\prime }\left(y\right){|}_{y=1}<0$ and Proposition 2 that, *S* > *S*′. It can be easily observed from the first order partial derivative of *Cei*, *w*, *Ie*, *De*, γ, *x*, *z*, *Fe*, and β for *S*′ that ∂*S*′/∂*Cei* > 0, ∂*S*′/∂*w* < 0, ∂*S*′/∂*Ie* < 0, ∂*S*′/∂*De* < 0, ∂*S*′/∂γ < 0, ∂*S*′/∂*x* > 0, ∂*S*′/∂*z* < 0, ∂*S*′/∂*Fe* < 0, and ∂*S*′/∂β < 0, respectively. Thus, *S*′ is positively correlated with *Cei* and *x*, and negatively correlated with *w*, *Ie*, *De*, γ, *z*, *Fe*, and β. The proof of Corollary 2.2 is complete.

### 3.3. Stability analysis for government

The expected revenue for the positive regulation (negative regulation) strategy implemented by the government is *Uz*(*U*_1 − *z*_), and its average expected revenue is Ū*z*:

(17) $$\begin{array}{l} Uz=xRg-yS-Cg+\left(1-x\right)\left[Fs-Dg+\left(1-y\right)Fe\right] \end{array}$$

(18) $$\begin{array}{l} U_{1-z}=xRg-yS+\left(1-x\right)\left[\left(1-y\right)\beta \left(Fs+Fe-Fg\right)-Dg\right] \end{array}$$

(19) $$\begin{array}{l} Ūz=zUz+\left(1-z\right)U_{1-z} \end{array}$$

The replicator dynamic equation and first-order derivative of government can be formulated as follows:

(20) $$\begin{array}{l} F\left(z\right)=dz/dt=z\left(Uz-Ūz\right)=z\left(1-z\right)P\left(x,y\right) \end{array}$$

(21) $$\begin{array}{l} F^{\prime }\left(z\right)=\left(1-2z\right)P\left(x,y\right) \end{array}$$

(22) $$\begin{array}{l} P\left(x,y\right)=\left(1-x\right)\left[Fs+\left(1-y\right)Fe-\left(1-y\right)\beta \left(Fs+Fe-Fg\right)\right]\\ \;-Cg \end{array}$$

It can be seen from Equations 20–22 that the government's strategic choice is influenced by that of the elderly care service providers and the elderly care service information platforms, as well as revenue and expenditure from various government departments. By using the stability theorem of the differential equation, the decision probability of government in a stable state must satisfy *F*(*z*) = 0 and *F*′(*z*) < 0.

**Proposition 3**. When *x* > *x*_0_, the government enforces negative regulations. When *x* < *x*_0_, the government enforces positive regulations. When *x* = *x*_0_, the stabilization strategy of the government cannot be determined. Here, the threshold is defined as follows:

$$\begin{array}{l} x_{0}=\left[Fs+\left(1-y\right)\left(Fe-\beta Fs-\beta Fe+\beta Fg\right)-Cg\right]\\ \;/\left[Fs+\left(1-y\right)\left(Fe-\beta Fs-\beta Fe+\beta Fg\right)\right]. \end{array}$$

**Proof of Proposition 3**. Because ∂*P*(*x, y*)/∂*x* < 0, *P*(*x, y*) is a decreasing function about *x*. When *x* > *x*_0_, we have that *P*(*x, y*) < 0, *F*(*z*)|_*z* = 0_ = 0, $F^{\prime }\left(z\right){|}_{z\;=\;0}<0$, and then *z* = 0 has stability. When *x* < *x*_0_, it follows that *P*(*x, y*) > 0, *F*(*z*)|_*z* = 1_ = 0, $F^{\prime }\left(z\right){|}_{z\;=\;1}<0$, and then *z* = 1 has stability. When *x* = *x*_0_, *P*(*x, y*) = 0, *F*(*z*) = 0 and *F*′(*z*) = 0, which cannot determine a stable strategy. The proof of Proposition 3 is complete.

Proposition 3 suggests that if the elderly care service providers provide high-quality services, the cost of positive regulations by government departments is not equal to the benefit. Thus, the government will switch from positive to negative regulations. When elderly care service providers offer low-quality services, the government faces significant social losses and the risk of accountability from higher levels and would prefer a positive regulatory strategy.

**Figure 4:**
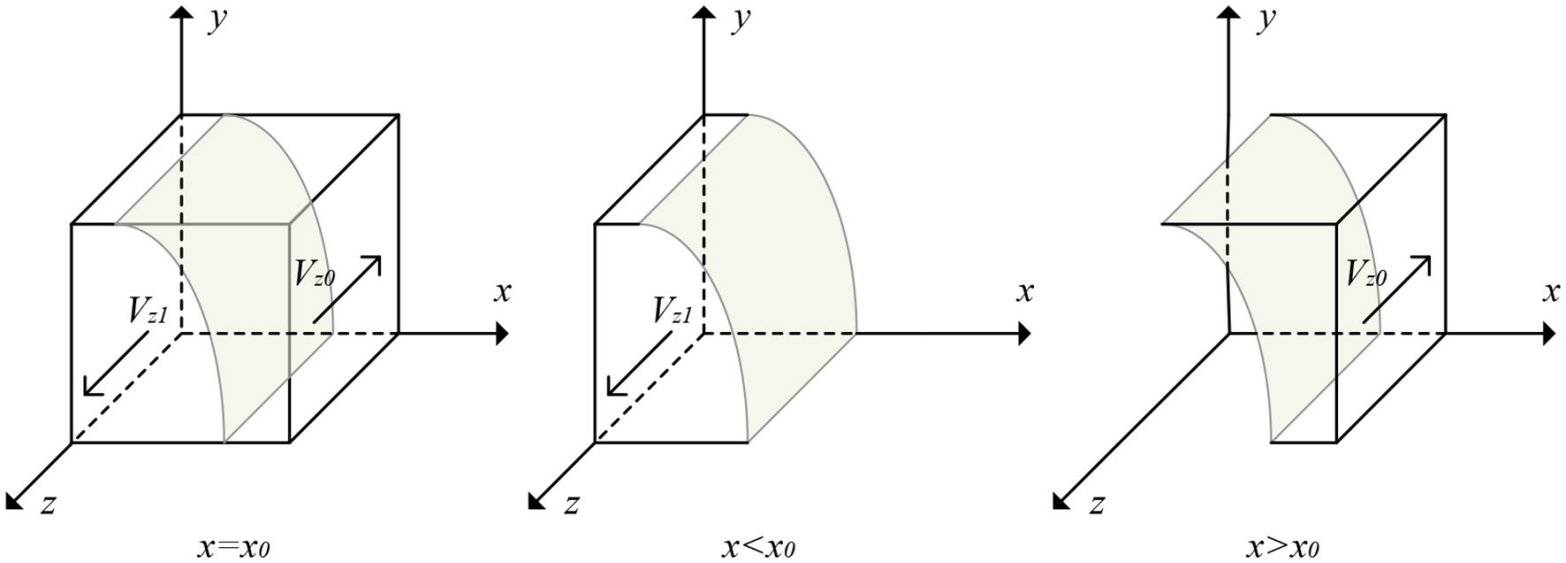
The evolutionary trajectory of the government's strategy.

From Proposition 3, the evolutionary trajectory of the government's strategy is obtained, as shown in Figure 4.

From Figure 4, we can see that the volumes of parts *Vz*0 and *Vz*1 are the probabilities of the government implementing negative and positive regulations. If we assume *m* = *Fe* − β*Fs* − β*Fe* + β*Fg*, then *x*_0_ = 1 − *Cg*/[*Fs* + (1 − *y*)*m*], and we have the following equation:

(23) $$\begin{array}{l} Vz1=\int_{0}^{1}\int_{0}^{1}1-Cg/\left[Fs+\left(1-y\right)m\right]dydz\\ \;=1-Cg\ln\;|\;1+m/Fs\;|\;/m \end{array}$$

(24) $$\begin{array}{l} Vz0=1-Vz1=Cg\ln\;|\;1+m/Fs\;|\;/m \end{array}$$

**Corollary 3.1**. The cost of positive regulations by the government negatively affects the probability of positive regulations when *Fg* > *Fs*. Here, *Fg* > *Fs* represents that the administrative penalties imposed by the superior government are greater than the revenue from penalties imposed by the government on the elderly care service providers.

**Proof of Corollary 3.1**. When *Fg* > *Fs*, we can see that *m* > 0, such that *Vz*1 = 1 − *Cg* ln (1 + *m*/*Fs*)/*m*. Using the first-order derivative, we can obtain that ∂*Vz*1/∂*Cg* = −ln (1 + *m*/*Fs*)/*m* < 0. When *Fg* < *Fs*, the probability of positive regulations by the government is influenced by a combination of factors, such as the rate of elderly people's complaints, the level of penalties imposed on the elderly service information platforms, and the level of policy subsidies for the elderly service information platforms. In this case, it should be discussed according to the specific situation. The proof of Corollary 3.1 is complete.

**Corollary 3.2**. If the administrative penalties *Fg* imposed by the superior government on the junior government are greater than a certain threshold *F*′*g*, it will choose a positive regulatory strategy. Under some scenarios, the superior government should increase the administrative penalties imposed on the junior government to urge him to regulate positively. These scenarios include an increase in the cost of positive regulations, a decrease in the revenue from penalties, a decrease in the complaint rate among elderly people, an increase in the probability of offering high-quality services, and an increase in positive return visit strategies. Here, the threshold *F*′*g* is defined as follows:

$$\begin{array}{l} F^{\prime }g=\left\{Cg-\left(1-x\right)\left[Fs+\left(1-y\right)\left(Fe-\beta Fs-\beta Fe\right)\right]\right\}\\ \;/\left(1-x\right)\left(1-y\right)\beta . \end{array}$$

**Proof of Corollary 3.2**. By using Proposition 3 $F^{\prime }\left(z\right){|}_{z\;=\;1}<0$, we get *Fg* > *F*′*g*. It follows from the first order partial derivative of *Cg*, *Fe*, *Fs*, β, *x*, and *y* for *F*′*g* that ∂*F*′*g*/∂*Cg* > 0, ∂*F*′*g*/∂*Fe* < 0, ∂*F*′*g*/∂*Fs* < 0, ∂*F*′*g*/∂β < 0, ∂*F*′*g*/∂*x* > 0, and ∂*F*′*g*/∂*y* > 0, respectively. Thus, *F*′*g* is positively correlated with *Cg*, *x* and *y*, and negatively correlated with *Fe*, *Fs* and β. The proof of Corollary 3.2 is complete.

### 3.4. Stability analysis for the elderly

The expected revenue for the online evaluation (offline evaluation) strategy chosen by the elderly is *Uw*(*U*_1 − *w*_), and its average expected revenue is Ū*w*:

(25) $$\begin{array}{l} Uw=xRo-Cw+\left(1-x\right)[\left(1-y\right)zIo+\beta \left(1-y\right)\left(1-z\right)\\ \;\left(Io-Co\right)+yIo-Do] \end{array}$$

(26) $$\begin{array}{l} U_{1-w}=xRo-Cm+\left(1-x\right)[\left(1-y\right)zIo+\beta \\ \;\left(1-y\right)\left(1-z\right)\left(Io-Co\right)+yIo-Do] \end{array}$$

(27) $$\begin{array}{l} Ūw=wUw+\left(1-w\right)U_{1-w} \end{array}$$

The replicator dynamic equation and first-order derivative for elderly people can be formulated as follows:

(28) $$\begin{array}{l} F\left(w\right)=dw/dt=w\left(Uw-Ūw\right)=w\left(1-w\right)\left(Cm-Cw\right) \end{array}$$

(29) $$\begin{array}{l} F^{\prime }\left(w\right)=\left(1-2w\right)\left(Cm-Cw\right) \end{array}$$

Equations 28 and 29 imply that the strategy of elderly people mainly depends on the cost of online and offline evaluations. Using the stability theorem of the differential equation, the decision probability of the elderly in a steady state must satisfy *F*(*w*) = 0 and *F*′(*w*) < 0.

**Proposition 4**. When the cost of the online evaluation is higher than that of offline evaluations, i.e., *Cm* − *Cw* < 0, elderly people will choose offline evaluations. When the cost of the online evaluation is lower than that of offline evaluation, i.e., *Cm* − *Cw* > 0, elderly people will choose online evaluation. When the cost of the online evaluation is equal to the cost of offline evaluations, i.e., *Cm* − *Cw* = 0, the stabilization strategy for elderly people cannot be determined.

**Proof of Proposition 4**. If *Cm* − *Cw* < 0, then *F*(*w*)|_*w* = 0_ = 0 and $F^{\prime }\left(w\right){|}_{w=0}<0$. Furthermore, *w* = 0 has stability. If *Cm* − *Cw* > 0, *F*(*w*)|_*w* = 1_ = 0 and $F^{\prime }\left(w\right){|}_{w=1}<0$, then *w* = 1 has stability. If *Cm* − *Cw* = 0, then *F*(*w*) = 0, *F*′(*w*) = 0, and no stable strategy for elderly people can be identified. Proposition 4 is complete.

Proposition 4 suggests that the strategy choice of elderly people mainly depends on the cost of offline and online evaluations. Elderly people will always choose the evaluation method with the lowest cost as their optimal strategic choice. Therefore, the government should urge the relevant units to provide the elderly with operation training for software for elderly services. This training would reduce the perceived cost of online evaluations for elderly people and thus encourage them to conduct them.

## 4. Stability analysis of strategy combinations

To explore the evolutionary stability strategies (ESS) and the conditions that need to be satisfied to reach the corresponding ESS, the stability of the system's strategy combinations needs to be analyzed. As is well known, the stable solution in a multi-group evolutionary game is a strict Nash equilibrium, i.e., a pure strategy equilibrium. Thus, Lyapunov's first method was used to analyze the stability of 16 pure strategy equilibrium points satisfying *F*(*x*) = 0, *F*(*y*) = 0, *F*(*z*) = 0, and *F*(*w*) = 0. The corresponding Jacobian matrix is stated as follows:

(30) $$\begin{array}{l} J=\left[\begin{array}{cccc} \partial F\left(x\right)/\partial x & \partial F\left(x\right)/\partial y & \partial F\left(x\right)/\partial z & \partial F\left(x\right)/\partial w\\ \partial F\left(y\right)/\partial x & \partial F\left(y\right)/\partial y & \partial F\left(y\right)/\partial z & \partial F\left(y\right)/\partial w\\ \partial F\left(z\right)/\partial x & \partial F\left(z\right)/\partial y & \partial F\left(z\right)/\partial z & \partial F\left(z\right)/\partial w\\ \partial F\left(w\right)/\partial x & \partial F\left(w\right)/\partial y & \partial F\left(w\right)/\partial z & \partial F\left(w\right)/\partial w \end{array}\right] \end{array}$$

### 4.1. Stability analysis in offline evaluation of elderly people

When the cost of online evaluations for elderly people is higher than that of offline evaluations, there are eight equilibrium points in the replicated dynamic system. The stability analysis of the equilibrium points is shown in Table 2.

**Table 2:** Stability analysis in the offline evaluations of elderly people.

**Equilibrium point**	**Eigenvalue λ_1_, λ_2_, λ_3_, λ_4_**	**Symbol judgment**	**Stability judgment**
*E*_1_(0, 0, 0, 0)	*Isγ* + *Csl* − *Csh* + *Dsγ* + β*Fs* + β*Io*, *Ieγ* − *Cei* + *S* + *Deγ* + β*Fe*, *Fs* + *Fe* − β*Fs* − β*Fe* + β*Fg* − *Cg*, *Cm* − *Cw*	× × × −	ESS when λ1 < 0, λ2 < 0 and λ3 < 0 are satisfied
*E*_2_(1, 0, 0, 0)	*Csh* − *Csl* − *Isγ* − *Dsγ* − β*Fs* − β*Io*, *Ieγ* + *Deγ* + *S* − *Cei*, −*Cg*, *Cm* − *Cw*	× × −−	ESS when λ1 < 0 and λ2 < 0 are satisfied
*E*_3_(0, 1, 0, 0)	*Isγ* + *Csl* − *Csh* + *Dsγ* + *Io*, *Cei* − *Ieγ* − *Deγ* − *S* − β*Fe*, *Fs* − *Cg*, *Cm* − *Cw*	× × × −	ESS when λ1 < 0, λ2 < 0 and λ3 < 0 are satisfied
*E*_4_(0, 0, 1, 0)	*Isγ* + *Fs* + *Csl* − *Csh* + *Dsγ* + *Io*, *Ieγ* − *Cei* + *Deγ* + *S* + *Fe*, β*Fe* + β*Fs* + *Cg* − *Fs* − *Fe* − β*Fg*, *Cm* − *Cw*	× × × −	ESS when λ1 < 0, λ2 < 0 and λ3 < 0 are satisfied
*E*_5_(1, 1, 0, 0)	*Csh* − *Csl* − *Isγ* − *Dsγ* − *Io*, *Cei* − *Ieγ* − *Deγ* − *S*, −*Cg*, *Cm* − *Cw*	× × −−	ESS when λ1 < 0 and λ2 < 0 are satisfied
*E*_6_(0, 1, 1, 0)	*Isγ* + *Fs* + *Csl* − *Csh* + *Dsγ* + *Io*, *Cei* − *Ieγ* − *Deγ* − *S* − *Fe*, *Cg* − *Fs*, *Cm* − *Cw*	× × × −	ESS when λ1 < 0, λ2 < 0 and λ3 < 0 are satisfied
*E*_7_(1, 0, 1, 0)	*Csh* − *Csl* − *Isγ* − *Dsγ* − *Fs* − *Io*, *Ieγ* − *Cei* + *Deγ* + *S*, *Cg*, *Cm* − *Cw*	× × +−	US
*E*_8_(1, 1, 1, 0)	*Csh* − *Csl* − *Isγ* − *Dsγ* − *Fs* − *Io*, *Cei* − *Ieγ* − *Deγ* − *S*, *Cg*, *Cm* − *Cw*	× × +−	US

×, Uncertainty of positivity or negativity; +, Eigenvalues are positive; −, Eigenvalues are negativity; US, Unstable point.

It can be seen from Table 2 that there are two instability points when elderly people are evaluated offline. The remaining six equilibrium points can potentially become more stable when certain conditions are met. As the elderly care service providers in the four pure strategy equilibrium points *E*_1_, *E*_3_, *E*_4_, and *E*_6_ all choose to offer low-quality services, these four equilibrium points are then undesirable strategy combinations. Some measures should be taken to improve the reputational gains and losses, the penalties imposed by the government, the complaint rate of elderly people, and the amount of compensation given to elderly people to prevent these points from becoming ESS. Thus, the losses incurred by the elderly care service providers fully cover the cost reduction incurred when they provide low-quality services. For both points *E*_2_ and *E*_5_, the elderly care service providers choose to offer high-quality services. Thus, these two equilibrium points are desirable strategy combinations. When the expected revenue from supplying high-quality services is greater than that from supplying low-quality services, both *E*_2_ and *E*_5_ may become stable points in the system. Specifically, the system will eventually stabilize at *E*_2_ when the sum of the reputational gains and losses and operating subsidies earned by the elderly services information platforms is less than the cost of positive return visits. Conversely, the system will stabilize at *E*_5_.

For points *E*_2_ and *E*_5_, it can be seen that when the elderly care service information platforms choose a negative return visit, the absence of their supervision function will result in insufficient constraints on the elderly care service providers. However, this insufficient constraint may be made up by the penalties imposed by the government on the elderly care service providers when the elderly actively defend their rights. This is the reason a course on legal awareness is offered to elderly people.

### 4.2. Stability analysis in the online evaluation of elderly people

When the cost of online evaluations for elderly people is lower than that of offline evaluations, i.e., *Cm* − *Cw* > 0, there might be eight equilibrium points in the replicated dynamic system. The stability analysis of the equilibrium strategy combination is shown in Table 3. As can be seen from Table 3, there are also two unstable points *E*_15_ and *E*_16_, four undesirable equilibrium points *E*_9_, *E*_11_, *E*_12_, and *E*_14_ that may become ESS and two desirable equilibrium points that can become ESS. The analysis process is similar to that of the offline evaluation of elderly people.

**Table 3:** Stability analysis in the online evaluation of elderly people.

**Equilibrium point**	**Eigenvalue λ_1_, λ_2_, λ_3_, λ_4_**	**Symbol judgment**	**Stability judgment**
*E*_9_(0, 0, 0, 1)	*Is* + *Csl* − *Csh* + *Ds* + β*Fs* + β*Io*, *Ie* − *Cei* + *De* + *S* + β*Fe*, *Fs* + *Fe* − β*Fs* − β*Fe* + β*Fg* − *Cg*, *Cw* − *Cm*	× × × −	ESS when λ1 < 0, λ2 < 0 and λ3 < 0 are satisfied
*E*_10_(1, 0, 0, 1)	*Csh* − *Csl* − *Is* − *Ds* − β*Fs* − β*Io*, *Ie* + *De* + *S* − *Cei*, −*Cg*, *Cw* − *Cm*	× × −−	ESS when λ1 < 0 and λ2 < 0 are satisfied
*E*_11_(0, 1, 0, 1)	*Is* + *Csl* − *Csh* + *Ds* + *Io*, *Cei* − *Ie* − *De* − *S* − β*Fe*, *Fs* − *Cg*, *Cw* − *Cm*	× × × −	ESS when λ1 < 0, λ2 < 0 and λ3 < 0 are satisfied
*E*_12_(0, 0, 1, 1)	*Is* + *Fs* + *Csl* − *Csh* + *Ds* + *Io*, *Ie* − *Cei* + *De* + *S* + *Fe*, β*Fe* + β*Fs* + *Cg* − *Fs* − *Fe* − β*Fg*, *Cw* − *Cm*	× × × −	ESS when λ1 < 0, λ2 < 0 and λ3 < 0 are satisfied
*E*_13_(1, 1, 0, 1)	*Csh* − *Csl* − *Is* − *Ds* − *Io*, *Cei* − *Ie* − *De* − *S*, −*Cg*, *Cw* − *Cm*	× × −−	ESS when λ1 < 0 and λ2 < 0 are satisfied
*E*_14_(0, 1, 1, 1)	*Is* + *Fs* + *Csl* − *Csh* + *Ds* + *Io*, *Cei* − *Ie* − *De* − *S* − *Fe*, *Cg* − *Fs*, *Cw* − *Cm*	× × × −	ESS when λ1 < 0, λ2 < 0 and λ3 < 0 are satisfied
*E*_15_(1, 0, 1, 1)	*Csh* − *Csl* − *Is* − *Ds* − *Fs* − *Io*, *Ie* + *De* + *S* − *Cei*, *Cg*, *Cw* − *Cm*	× × +−	US
*E*_16_(1, 1, 1, 1)	*Csh* − *Csl* − *Is* − *Ds* − *Fs* − *S* − *Io*, *Cei* − *S* − *Ie* − *De*, *Cg*, *Cw* − *Cm*	× × +−	US

×, Uncertainty of positivity or negativity; +, Eigenvalues are positive; −, Eigenvalues are negativity; US, Unstable point.

By comparing the conditions of the corresponding stability points in the online and offline evaluations, it can be concluded that the online evaluation channel for the elderly can increase the reputation losses and gains of elderly care service providers and elderly care service information platforms. Specifically, compared with offline evaluations, online evaluations make it more difficult for the system to create undesirable stability points and more likely to create desirable stability points. This is due to the fact that, in the case of the online evaluation, an undesirable stability point requires a higher cost reduction for elderly care service providers to reach stability. However, a desirable stability point can be achieved with lower compensation, fewer complaints from elderly people, and fewer penalties imposed by government departments.

Therefore, community-based elderly service centers should be relied upon to train the elderly in the community to operate the software for elderly care services. The positive effects of online evaluations should also be publicized to reduce the perceived cost of online evaluations for elderly people. It is important for the healthy development of “Internet + Community Elderly Care”.

## 5. Simulation and analysis

### 5.1. System dynamics model construction

**Figure 5:**
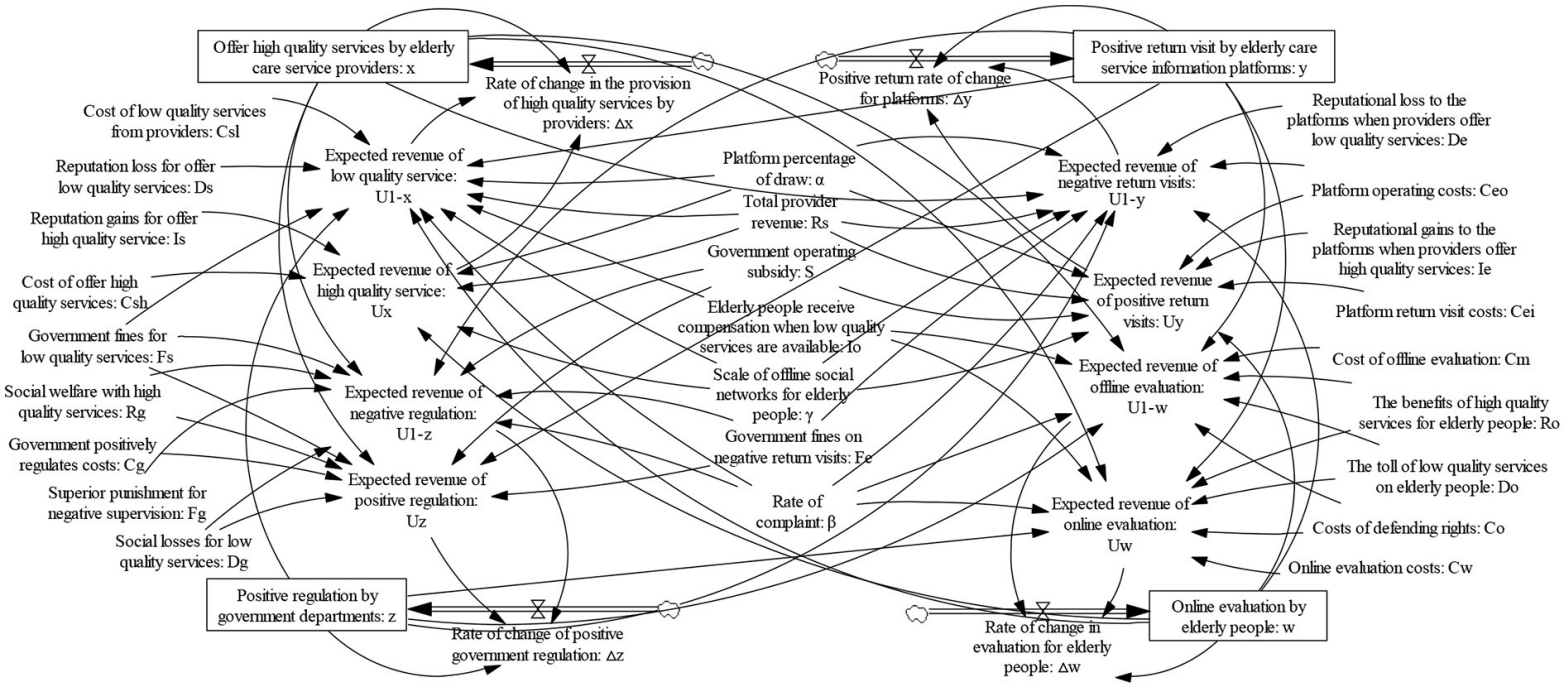
The SD model of “Internet + Community Elderly Care”.

System dynamics (52) (S.D.) is often applied to study complex systems and analyze evolutionary trends (53–55). Some authors recently used system dynamics to simulate and analyze evolutionary game problems (56–58). In this study, SD was incorporated into the study of the omnichannel feedback mechanism of elderly people to clearly depict the complex relationships and mechanisms of interaction among the subjects. Moreover, VENSIM 5.6b software was used for simulation analysis of the evolutionary game SD model, which involved government departments, elderly care service providers, elderly care service information platforms, and elderly people's participation. The proposed four-party evolutionary game SD model is shown in Figure 5. It consists of four sub-models: government departments sub-SD Model, elderly care service providers sub-SD Model, elderly care service information platforms sub-SD model and elderly people sub-SD Model. The four state variables, four rate variables, and a set of other intermediate and external variables in the model were determined by the model's construction. Furthermore, the functional relationships in the model were determined by the expectation function and the replicator dynamic equation.

### 5.2. Parameter setting and simulation analysis

To make the system stable to the ideal stability point, the following three conditions should be satisfied, i.e., *Cw* − *Cm* < 0, *Cei* − *Ie* − *De* < 0, and *Csh* − *Csl* − *Is* − *Ds* − *Io* < 0. Combined with the actual situation, the corresponding parameters are set as follows: *Rs* = 100, *Csh* = 50, *Csl* = 25, *Is* = 20, *Ds* = 22, *S* = 10, *Ceo* = 15, *Cei* = 5, *Ie* = 10, *De* = 12, α = 10%, β = 1%, γ = 5%, *Fs* = 20, *Fe* = 5, *Fg* = 200, *Rg* = 40, *Cg* = 10, *Dg* = 40, *Cm* = 10, *Cw* = 8, *Io* = 10, *Ro* = 20, *Do* = 30, and *Co* = 5. In addition, we assumed that *INITIAL TIME* = 0, *FINAL TIME* = 3, and *TIME STEP* = 0.0125.

#### 5.2.1. Influence of penalties and compensations on elderly care service providers

**Figure 6:**
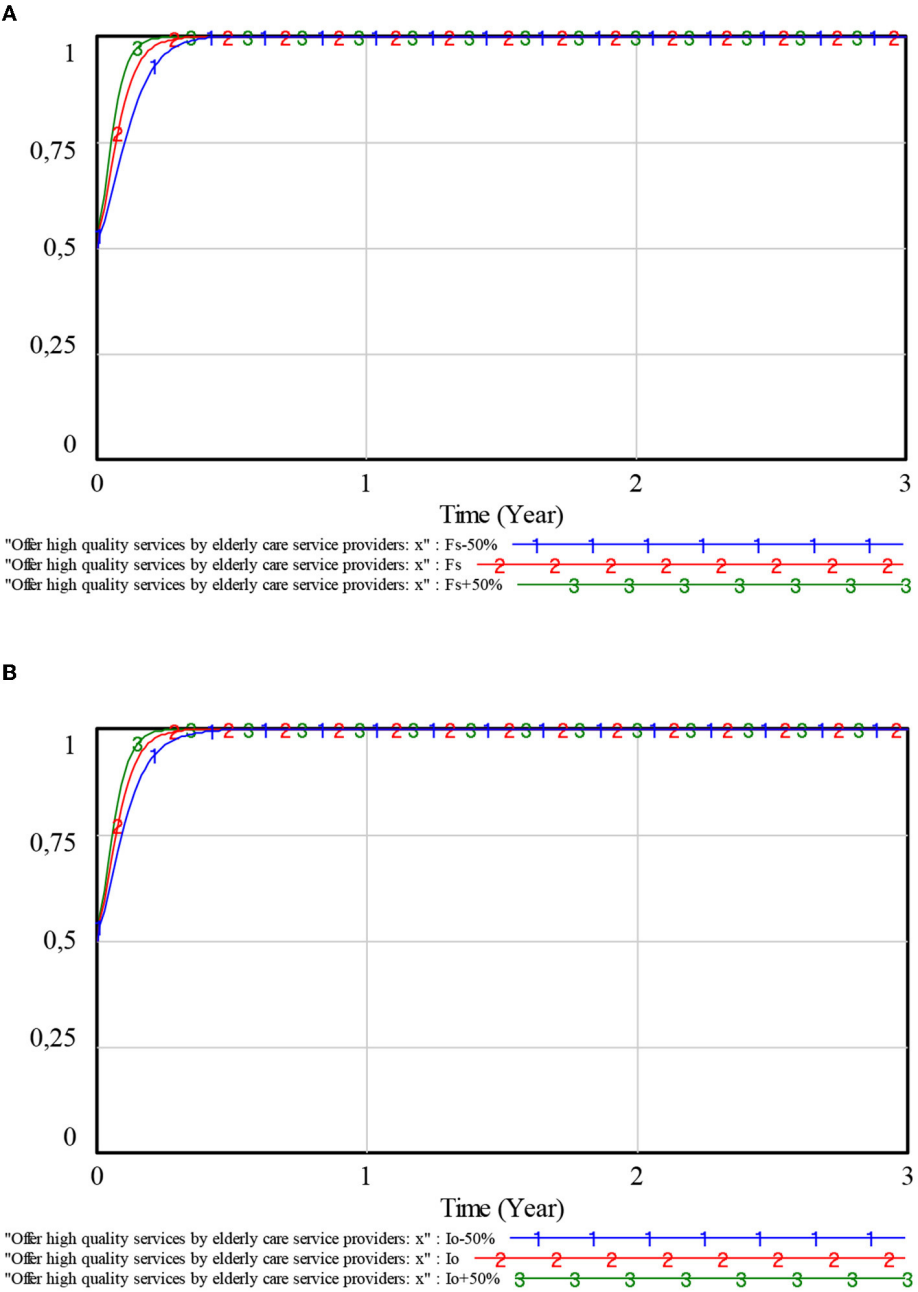
Influence of penalties and compensations on elderly care service providers. **(A)** Penalties. **(B)** Compensations.

To explore the effects of *Fs* and *Io* on the choice of elderly care service providers, the values of *Fs* and *Io* are set to fluctuate up and down by 50% around their initial values, respectively. The simulation results are shown in Figure 6.

From Figures 6A, B, it can be seen that, as the values of *Fs* and *Io* increase, the speed of the evolution of elderly care service providers to stable strategies has also increased to varying degrees. The greater the values of *Fs* and *Io*, the faster the elderly care service providers stabilize in providing high-quality services. This is due to the fact that the greater the values of *Fs* and *Io*, the greater the cost faced by the elderly care service providers for supplying low-quality services. Then, the elderly care service providers will tend to choose a strategy of providing high-quality services. In addition, when the values of *Fs* and *Io* change in the same proportion, *Fs* has an obvious impact on the evolutionary speed of elderly care service providers. With a 50% increase in both values of *Fs* and *Io* and the value of *Fs* by 50%, the elderly care service providers devised stable strategies faster.

On the contrary, with a 50% decrease in both values of *Fs* and *Io*, the elderly care service providers devise stable strategies faster with the value of *Io* decrease by 50%. Therefore, elderly care service providers are most sensitive to changes in penalties. Government departments can preferentially adjust *Fs* to guide the healthy development of the elderly market.

#### 5.2.2. Influence of penalties and subsidies on elderly care service information platforms

**Figure 7:**
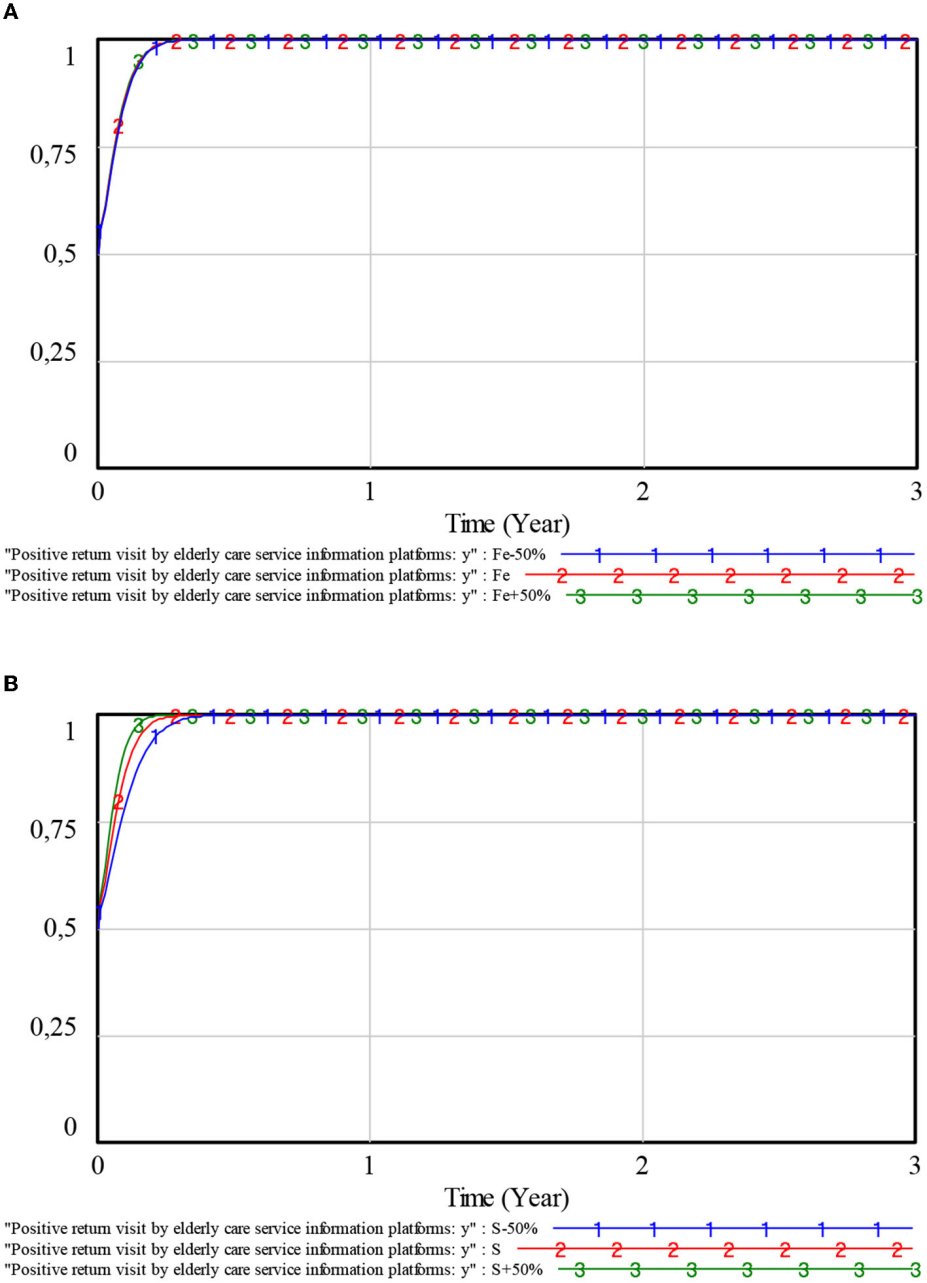
Influence of penalties and subsidies on elderly care service information platforms. **(A)** Penalties. **(B)** Subsidies.

To explore the influence of *Fe* and *S* on the choice of strategy for elderly care service information platforms, the values of *Fe* and *S* are set to fluctuate 50% up and down around their initial values, respectively. The simulation results are shown in Figure 7.

As can be seen from Figures 7A, B, changes in both values of *Fe* and *S* will have an impact on the evolutionary speed of the elderly care service information platforms. The greater the values of *Fe* and *S*, the faster the elderly care service information platforms stabilize in a positive return visit strategy. The smaller the values of *Fe* and *S*, the slower the elderly care service information platforms stabilize in a positive return visit strategy. However, the change in the value of *Fe* has little impact on the elderly care service information platforms, while the change in the value of *S* has a more significant impact on the elderly care service information platforms. It is because of this that the negative return visit behavior of the elderly care service information platforms is not illegal. Only when the negative return visit of the elderly care service information platforms makes the elderly care service providers provide low-quality services will the government punish the elderly care service information platforms due to inadequate management. The government encourages the active operation of elderly care service information platforms by providing subsidies. As a result, the elderly care service information platforms would choose a positive return visit strategy due to the government's adjustment of *S*.

#### 5.2.3. Influence of online evaluation on each player

**Figure 8:**
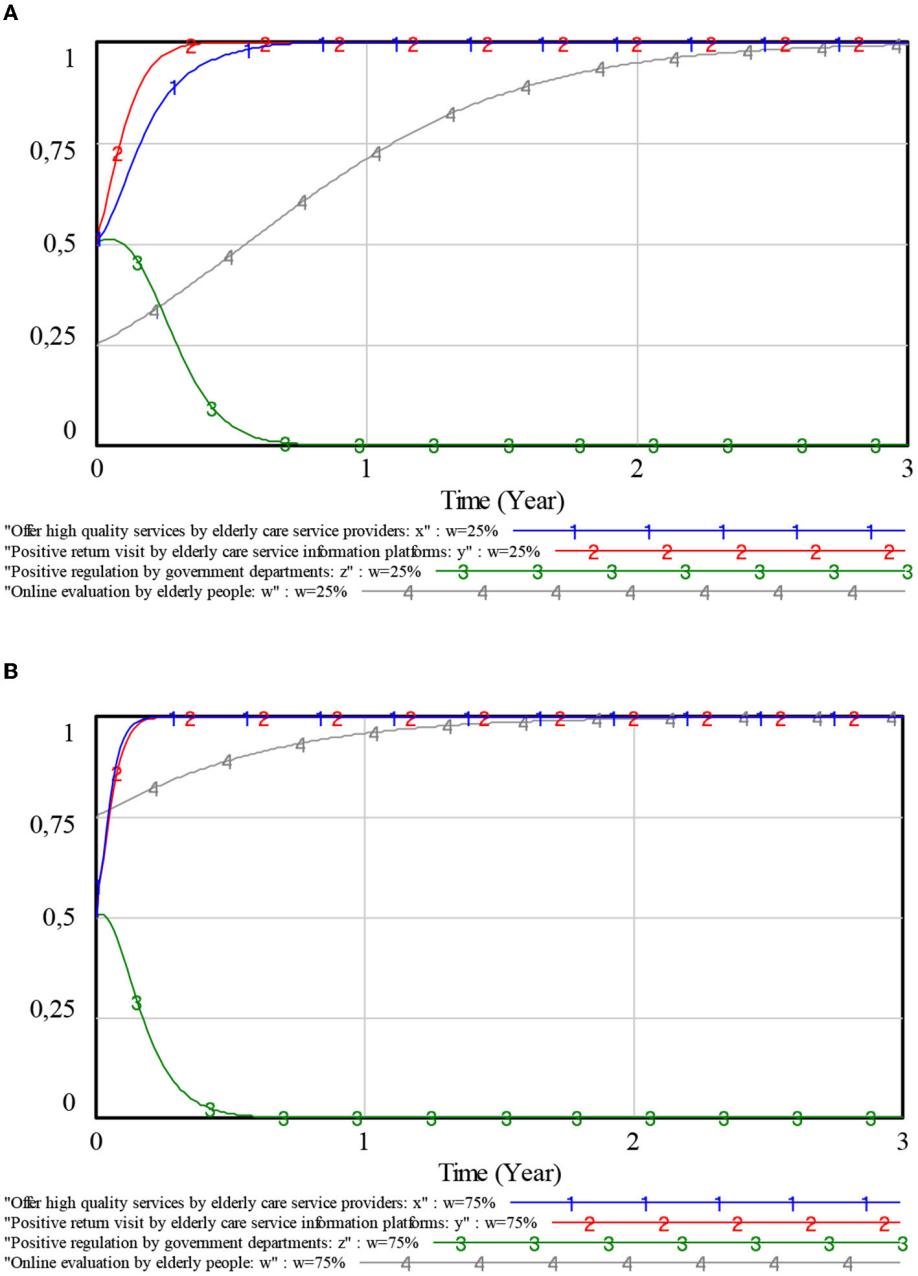
Influence of online evaluation on each player. **(A)** Online evaluation ratio 0.25. **(B)** Online evaluation ratio 0.75.

With other initial parameters unchanged, we observed that *w* = {0.25, 0.75}. The impact of the proportion of elderly people's online evaluations on the evolution of the decision-making behaviors of the government, elderly care service providers, and elderly care service information platforms is explored. The results are shown in Figure 8.

It can be seen from Figure 8 that with an increase of *w*, elderly care service information platforms and elderly people, evolve to a stable strategy of an active return visit and online evaluation emerged more quickly. At the same time, the evolution of elderly care service providers to provide high-quality services has accelerated due to the positive influence of behavior strategies adopted by elderly people and elderly service information platforms. In this case, the government will relax regulations and the tendency to increase negative regulations will rise. Therefore, online evaluation has a more positive effect on the regulatory system than offline evaluations. Some measures should be taken to increase the proportion of online evaluations of elderly people to promote the positive development of the whole “Internet + Community Elderly Care” system.

#### 5.2.4. Influence of reputational gains or losses on each player

**Figure 9:**
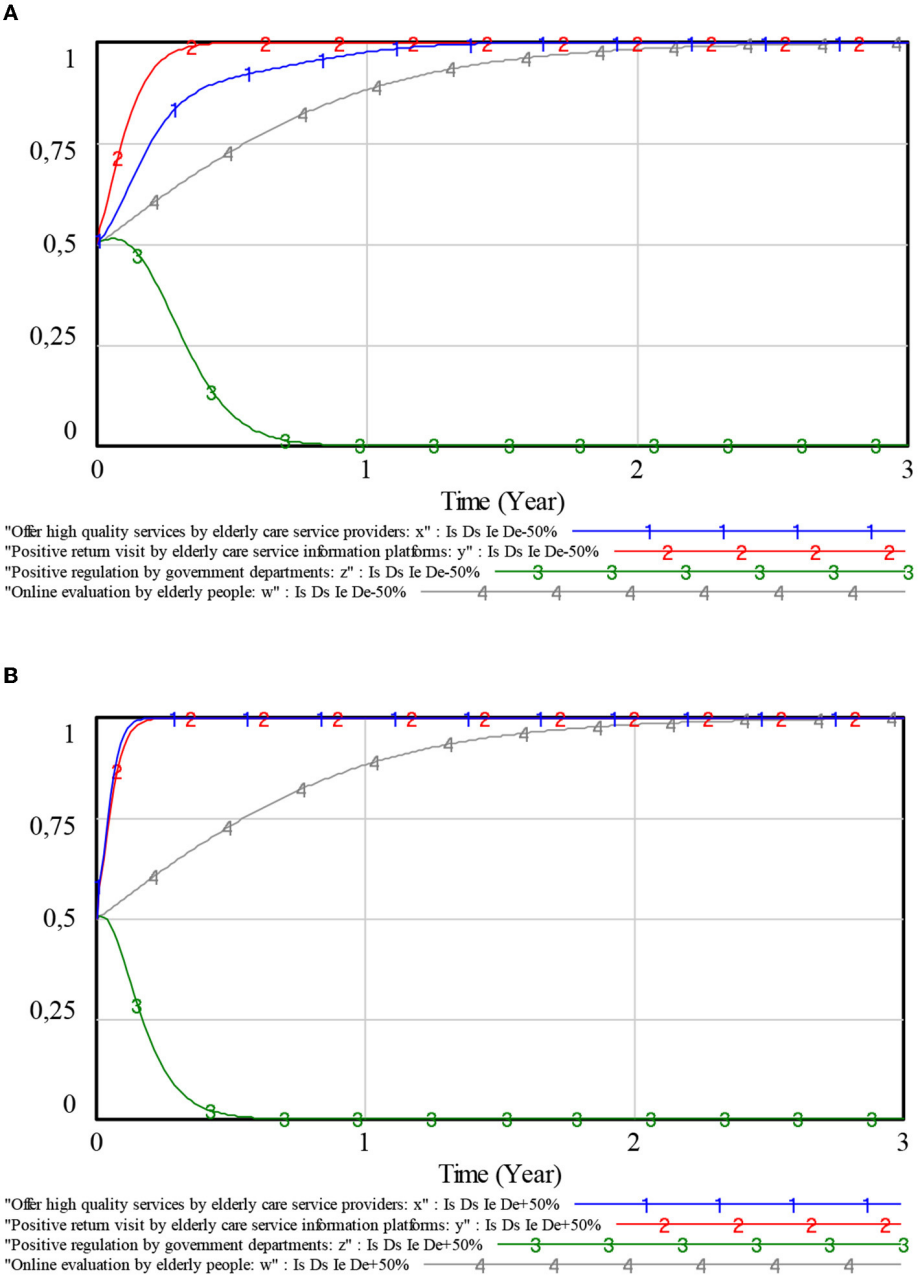
Influence of reputational gains or losses on each player. **(A)** Reputation gains or losses moved down by 50%. **(B)** Reputation gains or losses moved up by 50%.

To explore the impact of reputational losses and gains on the strategic choice of each player, we allowed the values of *Is*, *Ds*, *Ie*, and *De* to move up and down by 50% around their initial values. The results are shown in Figure 9.

As can be seen from Figure 9, changes in the values of *Is*, *Ds*, *Ie*, and *De* have a significant impact on the elderly care service information platforms, elderly care service providers, and the government. When the values of *Is*, *Ds*, *Ie*, and *De* are relatively low, the evolutionary speed of the elderly care service information platforms, elderly care service providers, and government to a stable state is relatively slow. With an increase in the values of *Is*, *Ds*, *Ie*, and *De*, the time to reach a steady state will be shortened for elderly care service providers and elderly care service information platforms. Since the elderly care service information platforms and the elderly care service providers have chosen positive behavior strategies, the government has relaxed the regulations and become faster at imposing regulations. This implies that an effective reputation mechanism can partially replace the government's regulations and reduce the regulatory pressure on the government. Therefore, establishing an effective reputation mechanism will be critical for the development of “Internet + Community Elderly Care.”

#### 5.2.5. Influence of complaint rate on each player

**Figure 10:**
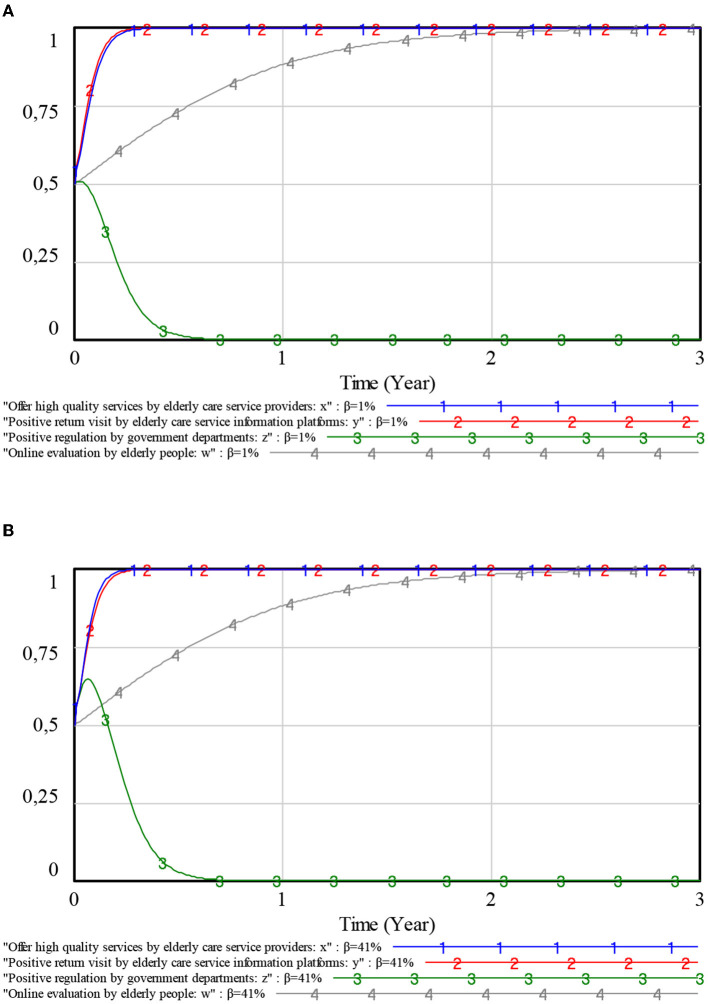
Influence of complaint rate on each player. **(A)** Complaint rate 0.01. **(B)** Complaint rate 0.41.

With the complaint rate β of the elderly being {0.01, 0.41}, the evolution of the decision-making behaviors of each player was explored. The simulation results are shown in Figure 10.

As can be seen in Figure 10, a change in the complaint rate β of elderly people has an impact on the strategic choices of elderly care service providers and government departments. However, there is essentially no impact on the strategic choices of elderly care service information platforms and elderly people. For the government, the higher the complaint rate β, the greater the fluctuation of their strategic choice toward positive regulations and the slower the evolution of their strategic choice toward negative regulations. For elderly care service providers, the higher the complaint rate β, the higher the possibility of providing high-quality services consistently. These suggest that complaints from elderly people can encourage both the government and elderly care service providers to choose positive behavioral strategies because there is a higher probability of being penalized. Therefore, it is vital to raise awareness about complaints and the rights of elderly people.

## 6. Discussions

This study presented an evolutionary game model to discuss “Internet + Community Elderly Care.” The stability of each player's strategy and the stability conditions of the replicated dynamic system's strategy combination were analyzed. The effects of changes in each key element on the strategic choices of the relevant players were also simulated in conjunction with system dynamics theory. The main suggestions of the study are proposed as follows:

First, online evaluations of elderly people have more positive effects on the regulatory system than offline ones. It is more difficult for the system to develop an undesirable stability point than a desirable stability point. Moreover, the proportion of elderly people evaluated online is increasing. The elderly care service providers will be more inclined to provide high-quality services, and the elderly care service information platforms will be more inclined to make return visits. Therefore, it is important to expedite the infrastructure construction of “Internet + Community Elderly Care.” However, elderly people need to be trained. The goal is to ultimately reduce the perceived cost of online evaluation for elderly people.

Second, the Omni-feedback mechanism for elderly people can effectively curtail the speculative behavior of elderly care service providers and elderly care service information platforms. It includes platform return visits, elderly people's online-offline evaluations, and complaint behaviors. Legal awareness seminars for elderly people can raise awareness of the elderly people's online and offline evaluations and complaint behaviors. Subsidized phone bills, training, and green channels for elderly people can reduce the cost of evaluations and complaints. Media participation or platform publicity can also improve the influence of feedback from elderly people. These measures can enhance the feedback mechanism for elderly people.

Finally, there are thresholds for the government to punish the elderly care service providers and subsidize the elderly care service information platforms. Only when those thresholds are reached can the government's punishment and subsidy policies be effective. The government should design the level of penalties imposed on the elderly care service providers with reference to their cost savings, reputational gains and losses, level of compensation, and complaint rate. More importantly, the government should dynamically adjust penalties. Similarly, the government can appropriately increase the operating subsidies for elderly care service information platforms to guide them in choosing a strategy for positive return visits.

## 7. Conclusions

The dual identity of the elderly as consumers and supervisors plays an important role in the supervision of the quality of elderly services. To achieve effective supervision of the quality of elderly services, it is important to boost the willingness of the elderly to participate in online evaluations, establish an effective reputation mechanism, and improve the rate of complaints from elderly people. These measures can curtail the speculative behavior of elderly care service providers while also reducing the pressure of supervision by the government. The government prioritizes penalties for elderly care service providers and subsidies for elderly care service information platforms that can effectively guide them. In order to maintain the effectiveness of government regulations, these penalties and subsidies should also be dynamically adjusted based on the actual situation.

Although the results in this study are rational and significant, there are still some limitations to the detailed description of the elderly people's feedback mechanism. Feedback from the elderly is not only differently influenced by the channel of feedback but is also affected by the emotions of the elderly or/and the induced evaluation behavior of the service providers. These influences may distort the content of the elderly people's feedback. Therefore, future research should consider real and distorted evaluations as a strategy for elderly people. Furthermore, elderly people should complain only when they receive real evaluations. Considering the variables of income and the cost of induced evaluations, the trend of behavior evolution and the influencing factors of participants merit further research.

## Data availability statement

The original contributions presented in the study are included in the article/supplementary material, further inquiries can be directed to the corresponding author.

## Author contributions

Conceptualization and methodology: YZ and QW. Software, validation, formal analysis, writing—original draft, and visualization: QW. Resources, writing—review and editing, and supervision: YZ and JL. Funding acquisition: YZ. All authors have read and agreed to the published version of the manuscript.
